# Understanding Sexual Safety in Residential Aged Care: A Workforce Perspective

**DOI:** 10.1155/jonm/7255533

**Published:** 2026-07-27

**Authors:** Robeena Emmanuel, Sheba Joseph, Bindu Narolil Mammen, Bindu Joseph

**Affiliations:** ^1^ Institute of Health and Wellbeing, Federation University Australia, P. O. Box 859, Berwick 3806, Victoria, Australia, federation.edu.au

## Abstract

**Background:**

Individuals living in Residential Aged Care Facilities (RACFs) have the right to sexual safety and to be protected from all forms of unwanted sexual behaviour. Emerging evidence suggests that sexual safety breaches can occur within aged care settings, placing vulnerable residents at risk. Aged care workers have a significant role in ensuring their sexual safety and protection.

**Purpose:**

This study aimed to explore the perspectives and experiences of aged care workers regarding the sexual safety of aged care residents.

**Methods:**

The study used an exploratory qualitative descriptive design. Data were collected from 22 participants (*n* = 14 nurses and *n* = 8 personal care workers) across various RACFs in Australia through semistructured interviews. Data were analysed using thematic analysis.

**Results:**

Five major themes derived from the study include the following: sexual safety matters: perspectives on sexual safety, dimensions of sexual vulnerability, it was just seen as behaviour: implications for identification and escalation, perceived barriers and building sexual safety through shared ownership.

**Conclusion:**

Findings highlight gaps in sexual safety knowledge, reporting practices and workforce preparation within residential aged care, with cultural and personal backgrounds influencing perceptions and responses to sexual safety incidents. Strengthening leadership, tailored mandatory sexual safety education and clear reporting pathways are essential to support a sexually safe environment while upholding residents’ sexual autonomy and rights.

**Implications for Nursing Management:**

Findings suggest that nursing management should prioritise the implementation of tailored sexual safety education, strengthen organisational reporting systems and support workforce capability in recognising and responding to sexual safety incidents. Leadership approaches that foster consistent reporting practices and uphold residents’ sexual autonomy may contribute to safer residential aged care environments.

## 1. Introduction

Sexual safety in Residential Aged Care Facilities (RACFs) refers to the protection and promotion of residents’ rights to dignity, autonomy, consent and freedom from sexual harm while acknowledging their right to intimacy, relationships and sexual expression [1–3]. Rather than relying solely on policies or compliance‐based training, achieving sexual safety within aged care settings requires a proactive, whole‐of‐system approach that safeguards residents from sexual abuse, harassment, coercion, intimidation and other sexually harmful or inappropriate behaviours that may compromise their well‐being, dignity, comfort, or sense of security [2, 4, 5]. Breaches of sexual safety are commonly defined as unwelcome behaviours or comments linked to sex, gender or sexual orientation that are experienced as offensive, humiliating or intimidating, such as sexual jokes, requests for sexual activity or nonconsensual physical contact [2]. Addressing such behaviours is central to ensuring sexual safety in RACFs.

The long‐term care setting of RACFs presents a unique environment, as these settings act as both residents’ homes and healthcare workplaces. This context makes sexual safety a particularly sensitive and complex issue, requiring staff to balance the responsibility of protecting residents from sexual abuse and harm while respecting their rights to autonomy, privacy, intimacy and sexual expression [6]. These responsibilities are further complicated by issues of consent and cognitive capacity, creating challenges in safeguarding residents’ well‐being while upholding their rights and maintaining appropriate professional boundaries for healthcare workers [2]. Sexual safety must therefore be understood within broader contextual factors, including cognitive impairment, dependence on care, communication barriers, personal histories and power imbalances and ongoing societal discomfort surrounding older adults’ sexuality [7–10]. Given these complexities, this study focuses specifically on sexual safety within RACFs.

Breaches of sexual safety in RACFs involve a spectrum of behaviours, ranging from sexual assault and sexual harassment to sexually disinhibited or other sexually harmful behaviours that may compromise a resident’s dignity, autonomy, comfort or sense of safety [11]. Sexual assault refers to any form of sexual contact that occurs without consent and is widely regarded as the most concealed, underrecognised and underreported type of elder abuse [12]. Sexual victimisation in RACFs is estimated to be twice (1.9% vs. 0.9%) as prevalent as in the community [13].

Perpetrators of sexual abuse in RACFs may include fellow residents, spouses, staff members, or visitors; in particular, male residents are reported to be the most common perpetrators [9, 12, 14]. Sexual offenders may exploit existing relationships with residents and staff, using trust and familiarity to gain access to vulnerable individuals. In some cases, staff perpetrators may deliberately seek opportunities to be alone with residents by volunteering for additional duties or less desirable shifts, behaviours that can be mistakenly perceived as dedication to their work [4].

As frontline practitioners, nurses and personal care workers (PCWs) are often responsible for monitoring, documenting and responding to residents’ behaviours, including those of a sexual nature. Despite this critical responsibility, evidence suggests that many staff members feel inadequately prepared to manage such situations effectively. An Australian study highlighted significant gaps in staff training and awareness regarding the prevention and management of sexual abuse in RACFs [15]. While there is a substantial and growing body of international literature on sexuality in residential aged care, much of this work has focused on residents’ sexual expression, sexual rights and organisational policy responses, including the management of sexual abuse. For example, recent research by Lee et al. [9] highlights public perceptions of sexual assault and its prevention in RACFs, reinforcing the emphasis on risk and safeguarding, including public awareness, staff training and policy‐level approaches. However, there is a lack of research exploring how aged care workers perceive, interpret, and respond to sexual safety concerns in real‐world care contexts. This gap is significant, as sexual safety issues are complex, context‐dependent, and frequently encountered in practice, requiring complex informed decision‐making. Addressing this gap is essential to support safe, consistent and person‐centred care that balances residents’ rights with their safety and well‐being.

Australia’s aged care sector is large and geographically dispersed, employing approximately 456,000 workers across around 736 approved providers managing about 2600 RACFs nationwide [16]. Most older Australians live in metropolitan areas, where most services and direct care workers are located, while regional and remote areas experience comparatively lower service and workforce availability. These patterns provide important context for understanding variability in workforce capacity, training access and safeguarding practices across settings. The Royal Commission into Aged Care Quality and Safety identified serious shortcomings in protecting residents, reporting an estimated 50 sexual assaults occurring weekly in Australian RACFs. The Commission called for urgent reforms, including improved reporting systems and stronger safeguards for residents [17]. Understanding aged care workers’ perspectives is therefore critical to informing practical, policy and leadership responses aimed at improving sexual safety in RACFs. The current study aimed to explore the perspectives and experiences of aged care workers regarding the sexual safety of aged care residents.

## 2. Methodology

### 2.1. Study Design

This study used an exploratory qualitative descriptive design to explore the perspectives of aged care workers registered nurses (RNs), enrolled nurses (ENs), and PCWs employed in RACFs concerning sexual safety. Sexual safety in aged care is a sensitive and complex issue, involving vulnerability, cognitive impairment, power dynamics, and organisational responsibility. An exploratory qualitative approach allowed for flexible, in‐depth exploration of aged care workers’ experiences without being restricted to a particular conceptual framework [18]. Additionally, an exploratory descriptive design was considered appropriate as there is limited published research exploring aged care workers’ experiences and perceptions of sexual safety among older adults in residential aged care settings. According to Hunter et al. [18], an exploratory qualitative design is appropriate when information is required directly from people who are personally experiencing the phenomenon under study. In this research, participants were professionals who directly provided care to residents in Australian aged care settings. The aim of this design is also to generate new insights and understandings grounded in participants’ experiences [18].

### 2.2. Ethical Considerations

Ethics approval for this study was obtained from the University Human Research Ethics Committee (HREC) (Approval number: 2025/183). Given the sensitive nature of the topic, particular attention and strategies were in place to protect participants from potential distress or harm. The study was advertised using flyers displayed in participating RACFs and via email. Nurses and carers who expressed interest in participating contacted members of the research team who had no direct professional relationship with either participants or aged care facility management. All interested participants were provided with a plain language information statement (PLIS), which outlined the study’s aim, what is involved, confidentiality and privacy information and participants’ rights. Participants provided verbal consent before the interview. They were also informed that participation was voluntary and that choosing not to participate or withdrawing from the study would not affect their employment. Due to the nature of the topic, participants were also informed that if disclosures indicated a current or serious risk of harm or a breach of legal obligations, the research team would follow mandatory reporting requirements.

### 2.3. Settings, Participants and Data Collection

The study was conducted across RACFs in Australia. The focus of this study was on staff experiences and perspectives rather than resident‐level demographic analyses or family responses. Participants were recruited using purposive convenience and snowball sampling methods, targeting aged care worker groups, including RNs, ENs and PCWs, who had direct experience providing care to residents in aged care settings. In the Australian aged care context, these staff groups comprise the core workforce responsible for delivering 24‐h direct care to older residents. Snowball sampling was also used, whereby initial participants assisted in recruiting additional participants through their professional and personal networks. The proportion of metropolitan and regional facilities represented in the sample was broadly consistent with the national distribution of RACFs in Australia.

Recruitment ceased at 22 participants in accordance with the principle of information power [19], whereby the sample size is determined by the adequacy and relevance of information for addressing the study aim. At this point, thematic saturation had also been achieved, with no substantially new themes or insights emerging from subsequent interviews. Data were collected through semistructured interviews. An interview guide was developed to support the interviews. The questions were informed by consultation with aged care nurse educators, academics with expertise in aged care, and literature. The interview guide explored aged care workers’ understandings of sexual safety, perceived risks, clinical responsibilities, organisational barriers and strategies for promoting safe care. All interviews were digitally audio‐recorded with participants’ consent and professionally transcribed verbatim. Experienced qualitative researchers with nursing or health backgrounds conducted interviews. The average interview duration was approximately 30 min.

Data were analysed using reflexive thematic analysis as described by Braun and Clarke [20–22]. Interview transcripts were transcribed verbatim and read repeatedly to ensure familiarity with the data. Initial codes were generated from the data and systematically organised to identify patterns of meaning. Codes were then grouped into potential themes, which were reviewed, refined and defined to ensure coherence and relevance to the study aim. The final themes were discussed with the research team and interpreted in relation to the study objectives and existing literature.

## 3. Results

As reported in Table 1, the participants were predominantly female (*n* = 20, 90.9%), reflecting the gender composition of the aged care workforce. The majority of participants were nurses (*n = *14, 63.4%), and others were PCWs (*n* = 8, 36.6%). Regarding organisational tenure, more than half of the participants (*n* = 15, 68.2%) reported over 5 years of experience in aged care settings, indicating a relatively experienced workforce. Most of the participants (*n* = 19) were employed in metropolitan RACFs. Participants represented diverse ethnic backgrounds, reflecting the multicultural nature of the Australian aged care workforce.

**TABLE 1:** Participant profiles.

Participant number	Position in the aged care	Sex	Years of experience	Nationality	Work location
P1	Personal Care Worker (PCW)	Female	7	India	Metropolitan
P2	PCW	Male	10	India	Metropolitan
P3	Registered Nurse (RN), Clinical Coordinator (CC)	Female	13	Sri Lanka	Metropolitan
P4	RN, CC	Female	7	India	Metropolitan
P5	RN	Female	8	Africa	Metropolitan
P6	RN	Female	5	Ireland	Metropolitan
P7	RN, CC	Female	5	India	Regional
P8	PCW	Female	35	Australia	Metropolitan
P9	PCW	Female	2	Australia	Metropolitan
P10	RN	Female	3	India	Metropolitan
P11	PCW	Female	10	India	Metropolitan
P12	RN	Female	17	India	Metropolitan
P13	RN	Male	2	India	Regional
P14	PCW	Female	15	Greek	Metropolitan
P15	PCW	Female	3	Australia	Metropolitan
P16	Enrolled Nurse (EN)	Female	5	India	Metropolitan
P17	EN	Female	15	South Africa	Metropolitan
P18	PCW	Female	1	Australia	Metropolitan
P19	RN, CC	Female	3	India	Metropolitan
P20	PCW	Female	4	India	Metropolitan
P21	RN	Female	5	Philippines	Regional
P22	RN	Female	23	Africa	Metropolitan

The analysis of the data revealed five main themes: sexual safety matters: perspectives on sexual safety, dimensions of sexual vulnerability, it was just seen as behaviour: implications for identification and escalation, perceived barriers and building sexual safety through shared ownership.

### 3.1. Theme 1: Sexual Safety Matters: Perspectives on Sexual Safety

This theme explores participants’ understanding of sexual safety within RACFs. Participants described sexual safety in ways that emphasised both the protection of residents’ autonomy and the prevention of harm.

For instance, one participant highlighted the importance of “creating a safe environment for residents where they feel safe. There should be no unwanted touch… if there is no consent” (P10), highlighting consent as a central principle in defining safety. Similarly, another participant framed sexually unsafe behaviour as exposure to “inappropriate sexual talk or touch that they do not want to participate in” (P13), reflecting a concern with both verbal and physical violations.

Together, these accounts suggest that participants conceptualise sexual safety primarily through the lens of consent, where the absence of willingness or choice transforms otherwise normative interactions into unsafe experiences. This framing indicates an understanding of sexual safety that is grounded in residents’ rights, bodily autonomy and protection from unwanted or intrusive behaviours.

Participants also recognised that sexual safety extended beyond overt sexual misconduct to include behaviours that may cause emotional discomfort or distress to residents. For example, one participant stated that “any activity that makes another person sexually uncomfortable is a safety concern” (P2).

This broader interpretation suggests that participants considered residents’ subjective experiences and emotional well‐being as important components of sexual safety within aged care settings.

In addition to protection from harm, participants highlighted the importance of respecting residents’ rights to sexual expression, intimacy and diverse sexual orientations. One participant noted that sexual safety involved ensuring “their bodies are protected and free from abuse; the safety of their sexual orientation and sexual expression is also important” (P15). Another participant stated, “It is also having sexual interaction with consent, and a staff member does not stop them from doing that but supports their sexual expression” (P22).

Overall, participants demonstrated a multidimensional understanding of sexual safety that incorporated protection from abuse, respect for consent, emotional well‐being, and support for residents’ sexual rights and expression within aged care environments.

### 3.2. Theme 2: Dimensions of Sexual Vulnerability

Participants identified several resident characteristics and conditions that they believed increased vulnerability to sexually unsafe behaviours within residential aged care settings. Cognitive impairment, particularly dementia, was consistently described as a significant risk factor. Participants explained that residents experiencing cognitive decline may have difficulty recognising inappropriate behaviours, communicating concerns or protecting themselves from exploitation.

“Dementia or cognitive impairment makes them vulnerable, for a family member or a staff member to exploit them, and they can’t even speak up for themselves sometimes” (P20). Another participant also stated, “We have a lot of residents with dementia… and we observe a lot of sexually inappropriate behaviours in the memory support unit… they can’t recollect when you ask them” (P22).

This highlights participants’ concerns regarding reduced capacity for self‐advocacy and increased dependence on others for protection and safeguarding.

Physical frailty and reduced mobility were also perceived to increase residents’ vulnerability to sexual safety risks. Participants described situations in which residents with significant physical limitations were unable to physically resist or avoid inappropriate behaviours. Participants highlighted how physical and communicative vulnerability shaped their understanding of sexual safety risks.

One participant explained, “I have non‐verbal, non‐ambulating residents, so they’re stuck in bed. So even if someone is trying to be inappropriate, they have no means of protecting themselves” (P8), emphasising how immobility and communication barriers can significantly limit residents’ ability to resist or report unwanted interactions. Another participant recalled intervening when “a male resident behaved in a sexually inappropriate manner toward a female resident who couldn’t move her arms” (P2), further illustrating how physical dependence can leave residents exposed to harm and reliant on staff for protection.

Together, these expressions suggest that participants perceive sexual safety through a lens of heightened vulnerability, where immobility, communication difficulties, and reliance on care increase the risk of unsafe situations. This reflects an understanding of sexual safety that extends beyond the management of behaviour to encompass recognition of residents’ reduced capacity to assert consent or protect themselves, highlighting the importance of proactive staff vigilance and intervention.

Gender was also identified as an important dimension of vulnerability. Some participants perceived female residents to be at greater risk of experiencing sexually inappropriate behaviours within RACFs.

“Female residents and immobile residents are more vulnerable” (P9), while another commented that “most of the time, sexual safety is a concern for females and exhibitors are mainly males” (P5).

Overall, participants described sexual vulnerability as multifactorial, with the interaction of cognitive impairment, physical frailty, communication limitations and gender increasing residents’ risk of experiencing sexually unsafe situations in RACFs.

### 3.3. Theme 3: “It Was Just Seen as Behaviour”: Implications for Identification and Escalation

Although participants generally demonstrated a clear understanding of sexual safety, individual judgement among aged care workers appeared to strongly influence how sexual safety incidents were identified, interpreted, escalated, and formally reported within RACFs. Participants described considerable variability in how staff responded to incidents, with some behaviours recognised as reportable safety concerns while others were minimised or interpreted differently depending on staff perceptions and workplace context.

“Any inappropriate behaviours, some gestures, it doesn’t have to be specifically physical, will be reported to the RN or management team for further review” (P20). These responses suggest that some staff viewed early reporting and escalation as important strategies for preventing potential harm and maintaining resident safety.

In contrast, other participants described selective reporting practices influenced by workload pressures, competing priorities, and perceptions of incident severity.

One participant reflected, “I think barriers like being understaffed, not having time to do things because you’re understaffed and under pressure to get all these tasks done and to do reporting” (P15), highlighting how staffing shortages and task‐driven environments can constrain thorough incident reporting. Similarly, another participant acknowledged, “We don’t report all of them. It’s like a 24/7 procedure, so we can’t just get everything recorded and escalated and solved; we just prioritise the stuff” (P13), suggesting that reporting decisions are often shaped by the need to triage and manage competing demands within continuous care settings.

Taken together, these accounts indicate that reporting practices are not solely determined by formal protocols but are actively negotiated within the realities of everyday clinical work. Participants’ emphasis on prioritisation and time constraints suggests that some incidents may be deemed less urgent or consequential and therefore not escalated.

This reflects a practice environment in which organisational pressures influence how sexual safety concerns are recognised and acted upon, potentially resulting in inconsistencies in reporting and gaps in documentation.

Another key issue identified by participants was the tendency for sexually inappropriate behaviours to be interpreted primarily as behavioural manifestations of dementia or cognitive impairment. Several participants described reporting concerns to RNs or senior staff but felt the incidents were subsequently minimised or reframed as expected behavioural symptoms associated with cognitive decline.

“I am comfortable reporting to the RN, but there were many occasions the RN said it is cognitive” (P3). Another participant reflected, “It was just seen as behaviour. When this old man tried to behave sexually inappropriately with a lady who was sitting nearby, she was in a condition that she couldn’t even react; I was told he has dementia, but I thought it was completely inappropriate” (P1).

Participants further expressed concern that categorising incidents as behavioural issues could contribute to the normalisation or reduced prioritisation of sexual safety concerns.

“Many incidents are categorised as behavioural, even if we report them, which will reduce the impact” (P2), while another stated that “sexually inappropriate actions are put under behavioural issues because most of the old people have cognitive issues. I have got experience with a couple of them touching other females inappropriately. It is all behavioural issues” (P1).

Overall, this theme highlights how staff interpretations and organisational responses can shape the identification and management of sexual safety concerns within RACFs. The findings suggest that when sexually inappropriate behaviours are primarily understood through a behavioural or dementia‐related lens, opportunities for timely recognition, reporting and intervention may be reduced.

### 3.4. Theme 4: Perceived Barriers

Participants identified several systemic and workforce‐related barriers that they believed hindered the provision of a sexually safe environment and affected the effective recognition, escalation and reporting of sexual safety incidents within RACFs.

#### 3.4.1. Systemic Barriers

Participants frequently raised concerns regarding the adequacy of training and preparation for aged care workers, particularly PCWs. Some participants perceived that the short duration of entry‐level training did not adequately prepare staff to care for highly vulnerable residents or manage complex sexual safety issues in practice.

“Three‐month training period for personal care workers is inadequate and insufficient preparation for caring for highly vulnerable adults” (P1). Another participant similarly identified “limited education of personal care workers and lack of sexual safety training are major issues in nursing homes” (P19).

These responses suggest that participants viewed workforce education as fundamental to building staff confidence and competence in identifying and managing sexually unsafe behaviours.

Workforce shortages and staffing instability were also perceived to affect staff preparedness and consistency of care. Participants explained that the frequent use of casual and agency staff reduced opportunities for comprehensive orientation to organisational policies, reporting pathways and safeguarding procedures. Participants perceived that inconsistent orientation and high workforce turnover could negatively influence staff understanding of sexual safety expectations and reporting responsibilities.

“Although there are policies and guidelines, not everyone gets oriented to them… because staffing shortages often require facilities to rely on casuals and agency nurses” (P5).

In addition, participants described organisational reporting systems as burdensome and difficult to navigate.

“The reporting process is complicated and time‐consuming” (P4), suggesting that procedural complexity itself may discourage timely documentation and escalation of incidents.

These findings indicate that organisational systems and workplace pressures may directly influence the consistency of sexual safety reporting practices within RACFs.

#### 3.4.2. Workforce‐Related Barriers

Participants also identified several workforce‐related factors that affected staff’s ability to recognise and appropriately respond to sexual safety concerns. Knowledge deficits, limited clinical experience, and a lack of confidence were commonly discussed as barriers to effective escalation and reporting.

“Now we have more staff after the Royal Commission, but if they don’t know when to escalate, no reporting occurs” (P5). Another participant described concerns regarding “inexperienced staff who don’t necessarily know what they’re looking for… also, language barrier issues can affect communication and incident recognition” (P8).

Cultural beliefs, personal attitudes, and discomfort discussing sexual matters were also perceived to influence staff responses to sexual safety incidents. Participants working within multicultural aged care environments explained that staff may hold differing understandings of sexuality, consent and acceptable behaviour based on their cultural backgrounds.

“Staff from different cultures may not feel comfortable talking about these issues” (P9), while another stated that “culture and personal beliefs are big barriers and that some staff experience fear of consequences of reporting” (P10).

Participants also noted the absence of standardised education to support consistent understandings of sexual safety across diverse workforce groups. As one participant explained, “everyone’s understanding of sexual safety thresholds is influenced by their culture, and there is no proper training or standardisation” (P5).

Overall, participants identified multiple interconnected systemic and workforce‐related barriers influencing the recognition, interpretation, escalation, and reporting of sexual safety incidents within RACFs. The findings suggest that organisational support, consistent education, clear reporting systems and culturally responsive workforce training may be important components in strengthening sexual safety practices in aged care settings.

### 3.5. Theme 5: Building Sexual Safety Through Shared Ownership

This theme captures participants’ perspectives on collective responsibility and the strategies required to promote and sustain sexual safety within RACFs. Participants described sexual safety as a shared responsibility among all aged care workers, rather than the responsibility of a single staff member or discipline. Many participants viewed protecting residents from sexually unsafe situations as an essential component of person‐centred care and everyday clinical practice.

“It is our responsibility to make sure they are sexually safe in their home” (P20), reinforcing the perception that RACFs should provide both physical and emotional safety for residents.

A strong emphasis was placed on the importance of ongoing education and training to strengthen staff knowledge, confidence, and consistency in responding to sexual safety concerns. Participants highlighted the need for training environments where staff feel comfortable discussing sensitive topics, particularly within culturally diverse workplaces where attitudes toward sexuality may vary.

“Proper training sessions for the staff where they feel comfortable because we have staff from different cultural backgrounds and with personal cultural values” (P10). “Sexual safety training, including early identification of warning signs, escalation and support, needs to be mandatory, just like falls training” (P3). Another participant stressed that “everyone should receive training in sexual safety and sexual safety reporting to support consistent practice across the workforce” (P15).

These responses suggest that participants viewed education as central to improving staff preparedness and reducing uncertainty around recognising and escalating sexual safety concerns.

Participants also discussed the importance of screening and risk assessment processes in preventing sexual safety incidents. Some participants believed that greater attention should be given to identifying histories of sexually inappropriate or harmful behaviours among residents.

“We should do a background check on the residents who had a history of any of these behaviours before coming into the facility” (P20).

Participants noted that sexual history‐taking and assessment of sexual safety risks were not routinely prioritised during admission processes, despite being perceived as important preventative measures.

At the same time, participants emphasised that promoting residents’ rights to intimacy, relationships or sexual expression is part of sexual safety and prevents sexual safety‐related incidents. Several participants described practices within their facilities that actively supported residents’ sexual autonomy while maintaining appropriate safeguards.

“We support sexual intimacy needs. We’ve had residents who have outsourced sex workers” (P8), while another explained “my facility developed sexual intimacy care plans and encourages that sexual intimacy where appropriate” (P7).

This theme highlights that participants viewed sexual safety as requiring collective ownership across all levels of aged care practice. The findings suggest that building sexually safe RACF environments involves coordinated organisational support, mandatory education, culturally sensitive communication, clear escalation pathways, proactive risk assessment and continued recognition of residents’ rights to sexual expression and intimacy.

## 4. Discussion

This study aimed to explore the perspectives and experiences of aged care workers regarding the sexual safety of aged care residents. This study also explored how they interpret, recognise and respond to such situations in everyday practice. The findings, therefore, reflect participants’ understandings and interpretations of sexual safety within their specific contexts, rather than representing fixed or universally accepted definitions of sexual safety or inappropriate behaviour. While participants generally demonstrated a broad understanding of sexual safety, including consent, autonomy and protection from verbal, physical and emotional harm, gaps in their knowledge and variability in the interpretation and management of sexual safety incidents remain. Despite the recognition that sexual safety is a shared responsibility, the influence of personal beliefs, cultural backgrounds, and limited education presents ongoing challenges.

Aged care residents are identified as highly vulnerable to sexual safety incidents due to various reasons; however, it remains the least reported form of elder abuse [12]. While there is an established body of literature on sexuality, intimacy, consent and sexual expression in residential aged care [4, 9, 23, 24], sexual safety has rarely been investigated as a distinct area of inquiry. All participants in the study displayed good insight into sexual safety incidents, identifying both verbal, physical and emotional factors affecting sexual safety. They emphasised that all residents, regardless of their gender, cognition and physical fragility, should feel safe and protected from any type of unwanted behaviours of a sexual nature. Participants also identified various vulnerability factors, such as cognitive impairment, being a woman, age, physical frailty and impaired communication, that could increase susceptibility to sexual harm. Multiple studies have highlighted that the factors like cognitive impairment, older females and physical frailty could increase the risk of sexual safety concerns in aged care settings [12, 14, 25]. In the current study, cognitive impairment was identified as a major factor, as it affects one’s ability to identify and report sexual safety breaches. Additionally, participants in the study identified male gender as a factor associated with exhibiting sexually inappropriate behaviour, as stated by Smith et al. [12].

The findings also highlight considerable variability in how sexual safety incidents are recognised, interpreted, escalated and reported within RACFs. Participants identified that personal beliefs, cultural backgrounds, varying levels of education, and organisational factors could influence staff responses to sexual safety concerns. While all participants reported feeling comfortable discussing sexual safety incidents, multiple participants identified an increasing tendency by the staff to classify certain acts of sexual safety incidents as just behaviours related to cognitive impairment or dementia. Several participants expressed concern that incidents categorised as “behavioural issues” were less likely to be formally escalated or recognised as sexual safety concerns. Emerging literature also suggests that aged care staff may interpret sexually inappropriate behaviours associated with dementia or cognitive impairment as disinhibition, behavioural disturbance, or a normal aspect of care work, which can contribute to the normalisation or underrecognition of sexual safety risks [23, 26]. Such interpretations may influence whether incidents are identified, documented or formally reported within residential aged care settings. Staff experience and personal judgment could also influence the interpretation of the warning signs of sexual safety breaches and escalation of such incidents. Supporting this, Lee et al. [27] argue that intentional injuries and bruising may resemble normal age‐related changes, thereby contributing to the potential underrecognition of physical indicators of sexual abuse among older adults. Another study identified significant underreporting of sexual abuse, which was attributed to barriers such as difficulties in recognising this form of abuse, limited awareness, and the influence of stigma [28]. Similarly, personal background, language barriers and cultural differences could influence one’s decision to report a sexual safety incident. Joseph et al. [29] also reported that language and cultural factors could influence aged care workers’ decision‐making capacity and the interpretation of policies.

Beyond personal factors, various organisational factors can influence the identification and escalation of sexual safety incidents. Participants in the present study described how staffing shortages and workload pressures could limit opportunities for proper orientation, supervision, reporting incidents and support for novice, casual and agency staff. Despite improvements in staffing ratios following the Royal Commission into Aged Care Quality and Safety’s recommendations, some participants reported that staffing remains insufficient on several occasions [17]. Again, the influence of adequate staffing and skill mix on the provision of safe care in aged care settings is emphasised in multiple studies [30–32]. Additionally, this study identified inconsistencies in documentation practices, reporting thresholds and escalation pathways as significant workplace challenges that may hinder staff members’ ability to consistently recognise, document, report and manage sexual safety incidents within RACFs. These findings align with Wright et al. [33], who similarly found that uncertainty around reporting thresholds and escalation pathways contributes to inconsistent responses to sexual safety incidents. Consistent with this, previous studies have also reported variability in organisational reporting practices and responses [26, 34], suggesting that such inconsistencies are a broader systemic issue.

While discussing various aspects of sexual safety, most participants in this study recognised the importance of supporting sexual intimacy and relationships within aged care settings. This aligns with Bauer et al. [35], who reported that sexuality in older age is often underacknowledged, poorly understood and inadequately addressed within healthcare contexts. Earlier research by Iversen et al. [36] found that some health professionals assumed older adults were no longer sexually active or interested in sexuality, reflecting more ageist or restrictive attitudes toward later‐life sexuality. Similarly, Myhre et al. [37] highlighted that supporting sexual expression can present ethical challenges for staff, particularly where cognitive impairment raises concerns regarding consent. Compared with these earlier findings, participants in the present study demonstrated greater recognition of residents’ rights to intimacy, companionship and sexual expression, suggesting a more contemporary and person‐centred understanding of sexuality in aged care. Distinctions between sexual expression, intimacy, behavioural manifestations and harmful or unsafe conduct are not always clear‐cut in practice. The findings highlight that staff frequently navigate these ambiguities when making decisions about recognition, escalation and reporting of sexual safety concerns. Importantly, this study does not position all sexual behaviours as inappropriate or harmful, nor does it suggest that all require escalation or formal intervention. Rather, it illustrates how aged care workers construct and negotiate understandings of sexual safety within routine care environments, including tensions between respecting residents’ autonomy and intimacy needs and fulfilling safeguarding responsibilities.

Consistent with previous studies [15, 38], participants in this study emphasised the critical importance of staff training and targeted education to enhance the early recognition of potential sexual safety breaches and improve staff knowledge and attitudes towards sexual safety. In this study, participants did not identify the existence of specific, dedicated training programmes on sexual safety or intimacy in residential aged care. Instead, they consistently reported that issues relating to sexual safety were addressed only as part of broader elder abuse, safeguarding or mandatory reporting education, rather than through explicit or targeted training content. Participants also highlighted the absence of routine assessment questions relating to previous sexually inappropriate behaviours or sexual safety risks during admission processes. Comprehensive screening and risk assessment of residents at admission were perceived by the participants as important preventive strategies.

The findings of this study also have important implications for nursing leadership and management within RACFs. Participants described sexual safety as requiring collective ownership across all levels of aged care practice, supported by clear organisational leadership and consistent safeguarding systems. Addressing sexual safety within RACFs requires leadership approaches that support evidence‐informed policies, clear reporting pathways, consistent documentation practices and ongoing workforce education. Nursing leaders play a critical role in fostering workplace cultures in which sexual safety concerns are recognised, appropriately escalated and addressed through coordinated safeguarding responses [23]. Organisational leadership is also essential in supporting culturally responsive education, role‐modelling appropriate professional behaviours and ensuring staff feel psychologically safe to report concerns without fear of negative consequences [23]. Incorporating sexual safety considerations into admission assessments, staff orientation processes, mandatory training programmes and ongoing professional development may strengthen prevention, early identification and organisational preparedness in responding to sexual safety risks within residential aged care environments.

### 4.1. Limitations and Directions for Future Research

This study has several limitations. As an exploratory qualitative study, the findings are not intended to be generalisable; however, they provide in‐depth insights into aged care workers’ perspectives and experiences of sexual safety within residential aged care settings. The sample was predominantly female and was from metropolitan areas (*n = *20), which may have influenced perspectives on sexual safety and associated experiences. Gendered perspectives may shape how sexual safety is understood and enacted in practice, and the underrepresentation of male participants limits exploration of potential differences in interpretation and response. Most participants were employed in metropolitan RACFs, which may not reflect the unique challenges and resource constraints experienced in rural or regional settings. Additionally, reliance on self‐reported experiences may have introduced social desirability response bias. To mitigate this, participants were assured of anonymity and confidentiality, participation was voluntary and interviews were conducted by researchers independent of participants’ workplaces. Nevertheless, the study provides rich descriptions of the research context, data collection and analytical processes, enabling readers to assess the transferability of the findings to similar settings. Future research should aim to include more diverse participant samples, particularly with respect to gender representation and geographic location, including rural and regional RACFs. Further studies could also use mixed‐methods or multiperspective designs (e.g., including residents, family members and managers) to better understand how sexual safety is constructed and managed across different stakeholder groups. In addition, research exploring how organisational culture, policy frameworks and regulatory environments shape staff interpretations and reporting practices would further strengthen the understanding of how sexual safety is operationalised in residential aged care.

## 5. Conclusion

Nurses and carers play a vital role in preventing, recognising, reporting, and managing sexual safety incidents in RACFs. However, these responsibilities should be balanced with respecting residents’ rights to consensual sexual expression, intimacy, privacy, dignity and autonomy. Supporting sexual safety therefore requires an approach that protects residents from harm while avoiding unnecessarily restrictive practices. This study highlights the influence of Australia’s multicultural aged care workforce on the sexual safety of residents, particularly in relation to how personal and cultural backgrounds may shape judgement and the threshold for reporting such incidents. These factors are important to consider when developing training and education programmes. Though most participants expressed the importance of the sexual safety of their residents, a gap in understanding and challenges in reporting were identified. A need for tailored mandatory sexual safety education is vital for improving the safety of residents. Despite staffing improvements following the Royal Commission into Aged Care Quality and Safety, staffing remains insufficient and continues to impact care delivery. To conclude, creating a sexually safe aged care environment requires shared responsibility, clear reporting pathways, culturally responsive training, robust admission screening processes and a continued commitment to respecting residents’ sexual autonomy.

## Funding

The authors received no specific funding for this work.

Open access publishing was facilitated by the Federation University Australia, as part of the Wiley‐Federation University Australia agreement via the Council of Australasian University Librarians.

## Disclosure

All authors have read and approved the final version of the manuscript.

## Ethics Statement

Ethics approval for this study was obtained from the Federation University Human Research Ethics Committee (HREC) (Approval number: 2025/183).

## Conflicts of Interest

The authors declare no conflicts of interest.

## Data Availability

The data that support the findings of this study are available on request from the corresponding author.
